# Evaluation of MMP-9, IL-6, TNF-α levels and peripheral blood mononuclear cells genes expression of MMP-9 and TIMP-1 in Iranian patients with coronary artery disease

**DOI:** 10.34172/jcvtr.2023.31844

**Published:** 2023-12-30

**Authors:** Tooran Akbari, Toktam Kazemi Fard, Reza Fadaei, Rahim Rostami, Nariman Moradi, Monireh Movahedi, Soudabeh Fallah

**Affiliations:** ^1^Department of Biochemistry, Faculty of Biological Sciences, North Tehran Branch, Islamic Azad University, Tehran, Iran; ^2^Department of Pharmacology, Vanderbilt University, Nashville, TN, USA; ^3^Department of Clinical Biochemistry, Faculty of Medicine, Iran University of Medical Sciences, Tehran, Iran; ^4^Department of Clinical Biochemistry, Faculty of Medicine, Kurdistan University of Medical Sciences, Sanandaj, Iran

**Keywords:** Coronary artery disease (CAD), Cytokines, Inflammation, Matrix metallopeptidase 9 (MMP-9), Tissue inhibitor of metalloproteinase (TIMP-1)

## Abstract

**Introduction:**

Coronary artery disease (CAD) is the main cause of death and is characterized by atherosclerosis in coronary arteries. Inflammation plays a crucial role in the progression and development of atherosclerosis.

**Methods:**

The present study consisted of 132 Iranian individuals who underwent coronary angiography, 65 patients with CAD, and 67 controls. The matrix metalloproteinase-9 (MMP-9), TNF-α, IL-6, and vitamin D serum levels were measured by the ELISA technique. The gene expression of MMP-9 and tissue inhibitors of metalloproteinase (TIMP-1) was estimated by real-time PCR assay.

**Results:**

A considerable increase in levels and PBMC gene expression of MMP-9 and serum levels of IL-6 and TNF-α were found in CAD patients compared with controls. A significant decrease was detected in vitamin D levels of CAD patients in comparison with controls. A considerable direct correlation was found between MMP-9 levels and MMP-9 and TIMP1 gene expression in CAD patients. MMP-9 levels positively correlated with LDL-C in CAD patients. The correlation between TIMP1 gene expression and IL-6 levels was also negatively significant. There were positive correlations between MMP-9 levels with IL-6 and TNF-α serum levels in CAD patients.

**Conclusion:**

This study showed that the interaction between MMPs, TIMP1, and cytokines could play a role in the pathogenesis of atherosclerosis. The present study suggested that high levels of TNF-α and IL-6 and vitamin D deficiency in our studied patients could disturb the MMP-9/TIMP-1 balance and lipid metabolism, leading to plaque formation/ rupture in predisposed CAD patients.

## Introduction

 Coronary artery disease (CAD) is a condition in which blood flow to the heart muscle is reduced due to the build-up of atherosclerotic plaque in the arteries of the heart. CAD is a major cause of morbidity and mortality globally, and its development is impacted by lifestyle, genetics, and the environment.^1-3^ Risk factors, including diabetes, dyslipidemia, hypertension, obesity, and inflammatory factors, contribute to vascular atherosclerosis and CAD development and progression. Inflammatory cytokines, matrix metalloproteinases (MMPs), and tissue inhibitors of metalloproteinases (TIMPs) are the main risk factors for CAD, as they contribute to plaque formation and atherosclerosis pathogenesis.^4,5^ However, the molecular mechanisms involved in developing cardiovascular and atherosclerosis diseases still need to be comprehended completely.

 Matrix metalloproteinase-9 (MMP-9), a gelatinase- type IV collagenase member of extracellular zinc-dependent endopeptidases with 92 KDa, degrades adhesion proteins and extracellular matrix (ECM). The activity of MMP-9 regulates by TIMP.^6,7^ According to research findings, MPP-9 has the potential to reduce plaque size, increase their susceptibility, and induce the infiltration of immune-inflammatory cells by acting on the basal membrane. This may facilitate angiogenesis by promoting the proliferation of endothelial cells.^5,6^ Furthermore, MMP-9 polymorphism, MMP-9/TIMP-1 imbalance and ECM turnover are considered the leading cause of coronary plaque instability and mortality.^8-10^

 The precise interaction between pro-inflammatory cytokines and MMP-9 is significant in atherosclerotic plaque formation and vascular lesions.^11,12^ Several pro-inflammatory cytokines, including tumor necrosis factor-α (TNF-α) and interleukin-6 (IL-6), are involved in atherosclerotic plaque formation and vascular/tissue lesion via overexpression of MMP-9, downregulation of TIMP-1 and LDL transcytosis.^13-16^ To our knowledge, both TNF-α and IL-6 possess the potential to activate transcription factors, most notably PPAR-γ and NF-κB pathways. This activation can trigger the transcytosis of LDL across the endothelium, thereby starting the progression of atherosclerosis. Furthermore, it can result in the activation of reactive oxygen species (ROS) and inflammasomes.^15,16^

 Emerging evidence has suggested that Vitamin-D (Vit-D) plays a crucial role in CAD aetiology, and Vit-D insufficiency is associated with CAD severity. Vitamin D plays a protective role in preventing atherosclerosis through its anti-inflammatory and anti-oxidant effects. It also regulates the growth, migration, differentiation, and immune system modulation of vascular cells, while inhibiting fibrotic signaling.^17,18^ Moreover, Vitamin D helps in regulating foam cell formation, ECM turnover, expression of MMPs and TIMPs, calcium concentration, and cardiac contractility, which results in a decrease in cardio-hypertrophy.^7-18^ Vit-D deficiency in heart diseases leads to inflammation and ECM remodeling by activating of inflammatory transcription factors such as *NF- κB and AP-1.*^19,20^

 According to recent findings, MMPs and inflammatory factors contribute to the development and progression of cardiovascular diseases. Hence, the present study aimed to explore the interrelationship between the lipid profile, Vit-D, Zn^2+^, Ca^2+^, TNF-α and IL-6 with PBMC gene expression of MMP-9, TIMP-1 and MMP-9 levels in patients with CAD and controls.

## Materials and Methods

###  Population

 A cohort of 132 Iranian subjects underwent a study at Hazrat-e-Rasoul Hospital during coronary angiography, at the Iran University of Medical Sciences. This study was conducted between April 2020 and February 2021 and using the confidence interval method, sixty-five patients with CAD and sixty-seven healthy controls were recruited. Inclusion criteria was CAD diagnosis by coronary angiography.

 The diagnosis of CAD is typically made by a cardiologist when an individual has at least one major coronary artery with stenosis greater than 50%. Conversely, healthy controls are identified from participants whose coronary arteries exhibit less than 25% stenosis.^19^

 All participants were subject to exclusion criteria, which included having a history of chronic diseases, chemotherapy, steroid hormone therapy, cancer, liver diseases, diabetes, renal failure and anti-inflammatory medication in the last six months. Additionally, the study did not include individuals with unstable angina, carotid plaque, a history of cardio-cerebrovascular disease, acute coronary syndrome, or peripheral artery disease as controls. The studied subjects’ medical history, demographic information, and medication consumption were collected by questionnaire. Anthropometric information was measured, including systolic blood pressure (SBP) and diastolic blood pressure (DBP), weight, height and body mass index (BMI). The study was approved by the Ethics Committee of Iran University of Medical Sciences, Tehran, Iran. (IR.IUMS.FMD.REC.1398.059). According to the Declaration of Helsinki, informed written consent was obtained from all participants (IR.IUMS.FMD.REC.1398.059).

###  Blood Collection and laboratory parameter measurements

 Fifteen mL Venous blood was collected following night fasting (8 to 12 hours) and stored at -80°C. The lipid profile, which is shown in Table 1 and the levels of fasting blood sugar (FBS) were measured (Pars Azmon kit, Iran).

**Table 1:** Anthropometric and laboratory profiles of study population

	**Control (n=67)**	**CAD (n=65)**	
	**Mean±SD**	**Mean±SD**	***P*** ** value**
Age (Years)	57.40 ± 8.29	60.56 ± 7.58	0.014
BMI (kg/m^2^)	26.23 ± 4.45	27.6 ± 4.24	0.050
SBP (mmHg)	129.54 ± 18.88	133.48 ± 30.60	0.333
DBP (mmHg)	81.6 ± 12.33	82.15 ± 15.84	0.809
FBS (mg/dL)	92.09 ± 10.47	95.23 ± 13.61	0.108
TG (mg/dL)	132.12 ± 47.67	140.86 ± 41.74	0.223
TC (mg/dL)	135.26 ± 28.16	138.11 ± 28.86	0.533
LDL-C (mg/dL)	68.31 ± 30.03	72.57 ± 22.36	0.317
HDL-C (mg/dL)	44.75 ± 8.62	38.86 ± 9.29	0.001
MMP-9 levels (ng/ml)	761.70 ± 230.25	1097.86 ± 364.76	0.001
TNF-α (pg/mL)	9.33 ± 3.85	12.39 ± 5.39	0.002
IL-6 (pg/mL)	5.41 ± 3.62	8.02 ± 5.67	0.002
MMP-9 gene expression	1 ± 0.14	1.11 ± 0.17	0.001
TIMP-1 gene expression	0.178 ± 0.039	0.182 ± 0.019	0.398
Zn^+2^ (µg/dl)	23.18 ± 1.36	23.61 ± 1.71	0.088
Ca^+2^ (mg/dl)	9.76 ± 1.55	9.20 ± 2.32	0.077
Vitamin D (ng/ml)	43.05 ± 22.57	28.74 ± 20.97	0.002

*P*<0.05 is statistically significant.

###  Serum TNF-α, IL-6 and MMP-9 assay

 The levels of serum MMP-9, IL-6 and TNF-α were assessed by ELISA method. Suggested manufacturer assay range for TNF-α was 15.6 - 1,000 pg/mL, for IL-6 was 7.8 pg/ml - 500 pg/ml, and for MMP-9 was 105.47 pg/ml - 6750 pg/ml (Abcam, USA; TNF-α Cat. No: ab181421, IL-6 Cat. No: ab178013, MMP-9 Cat. No: ab246539). Intra and inter assay for TNF-a, IL-6 and MMP-9 were 2.5%-3.1%, 2.1-2.4% and 2.5-6%.

###  PBMCs isolation and RNA-Extraction

**Figure 1:**
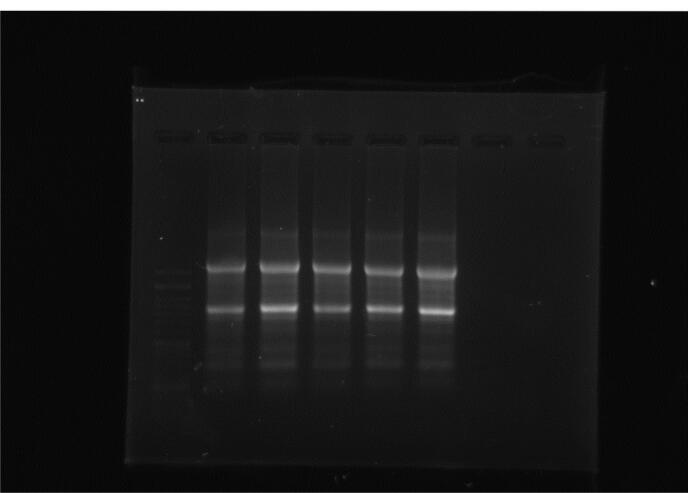
Electrophoresis of extracted RNA in 1.5% agarose gel

 As previously described, 10 mL whole blood was collected in test tubes containing EDTA for PBMC isolation by Ficoll-Hypaque density-gradient centrifugation.^21^ Extracting total RNA from cell lysates was done by miRNeas mini kit, following the instructions provided by the manufacturer (QIAGEN, USA, Cat. No. / ID: 74004). For evaluating the purity and concentration of extracted RNA, agarose gel electrophoresis (1.5%) (Figure 1) (Bio-Rad, USA, Cat. No: 161-0722) and the Nano-drop spectrophotometer were used (thermo-scientific, US).

###  Synthesis of cDNA and mRNA Quantitation

 To evaluate gene expression, the real-time PCR (RT-PCR) method was utilized. The process involved converting total RNA to cDNA and then running RT-PCR on an ABI-Step One system (Takara, Japan: Cat. #6110A and Applied Biosystems, USA). Real Plus 2x Master Mix Green in our RT-PCR was used, with one µg of RNA (Amplicon, Denmark: Cat. No.: A323402). To ensure accuracy, GAPDH was used as an internal control and designed specific primers (Table 2) for amplification of the studied genes.

**Table 2:** Specific primers for MMP-9, TIMP-1 and GAPDH genes

**Genes**	**Specific primers**
MMP-9	F: 5′ - CCTGGGCAGATTCCACT- 3′ R: 5′ - CCAAGTGTTCCGAGTAGTTTTGGA - 3′
TIMP-1	F: 5′ - ACTGCAGGATGGACTCTTGCA - 3′ R: 5′ - TTTCGAAGCCTTGGAGGAGCT - 3′
GAPDH	F: 5′ - CCCCTTCATTGACCTCAACTAC - 3ʹ R: 5′ - GATGACAAGTTCCCGTCT C - 3′

###  Statistical Analysis

 The statistical analysis was conducted using the SPSS software (V.20, IBM, Chicago, IL, USA). Results were presented as mean ± standard deviation and median values. Both parametric and non-parametric variables were analyzed using the student t-test and Mann-Whitney U test, respectively. To analyze categorical data, we conducted the χ2 test and presented the results in frequency and percentage. We utilized Pearson’s correlation coefficient or Spearmen’s correlation test to assess any connections between variables. The Receiver Operating Characteristic (ROC) was employed to evaluate the diagnostic ability of the variables of interest. A *P* value < 0.05 is considered significant.

## Results

###  Anthropometric and biochemical assessment

Table 1 shows that BMI, age, sex, SBP and DBP were similar in the CAD and control groups. Furthermore, Zn^+2^, FBS, TG, LDL-C, and TC levels did not show significant differences, while levels of Ca^2+^ and HDL-C levels were significantly lower in CAD than in controls.

**Figure 2:**
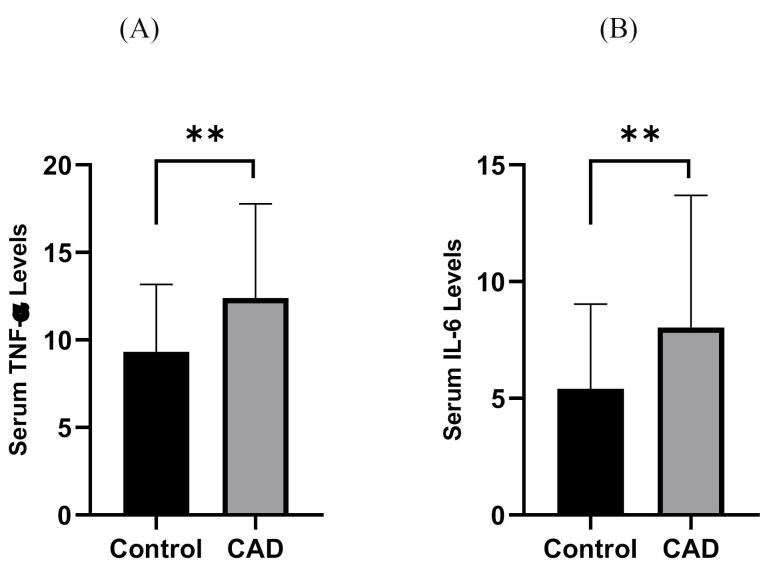
Comparison between (A) TNF-α and (B) IL-6 levels of CAD patients and controls **P < 0.01 CAD = Cardiovascular Disease

**Figure 3:**
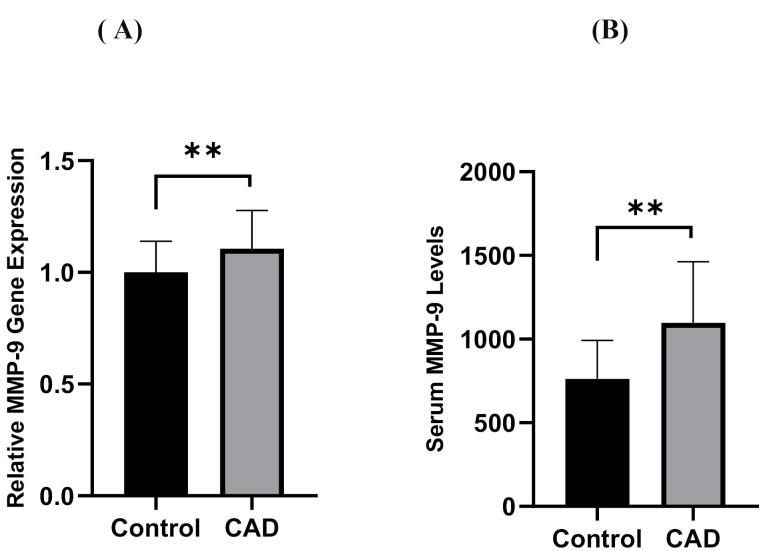
Comparison between serum concentration (A) PBMC gene expression of MM-P 9 and (B) of MMP- 9 levels between CAD patients and controls. CAD = Cardiovascular Disease, PBMC = Peripheral blood mononuclear cells **P < 0.01

**Figure 4:**
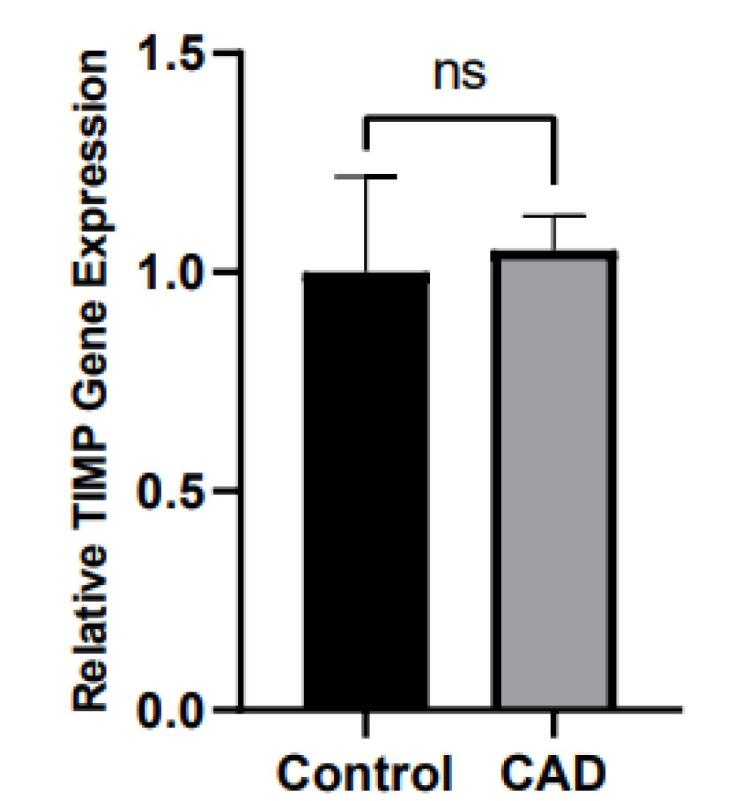
Comparison of PBMC gene expression of TIMP between CAD patients and controls CAD = Cardiovascular Disease, TIMP = Tissue Inhibitor of metalloproteinase

 TNF-α and IL-6 levels were significantly elevated in CAD patients compared to controls (12.39 ± 5.39 pg/mL vs. 9.33 ± 3.85 pg/mL and 8.02 ± 5.67 pg/mL vs. 5.41 ± 3.62 pg/mL, all *P* = 0.01, respectively), as shown in Figure 2A & 2B. Moreover, PBMC gene expression of MMP-9 and MMP-9 levels was significantly higher in CAD than in control (1.11 ± 0.17 vs. 1 ± 0.14 and 1097.86 ± 364.76 ng/ml vs. 761.70 ± 230.25 ng/ml; all *P* = 0.01, respectively) (Figure 3A & 3B). However, the two groups had no considerable change in TIMP1 gene expression (Figure 4). In CAD patients, circulating Vit-D levels were lower than the controls (29.58 ± 22.03 ng/mL vs. 44.26 ± 22.02, *P* = 0.0002, respectively).

**Figure 5:**
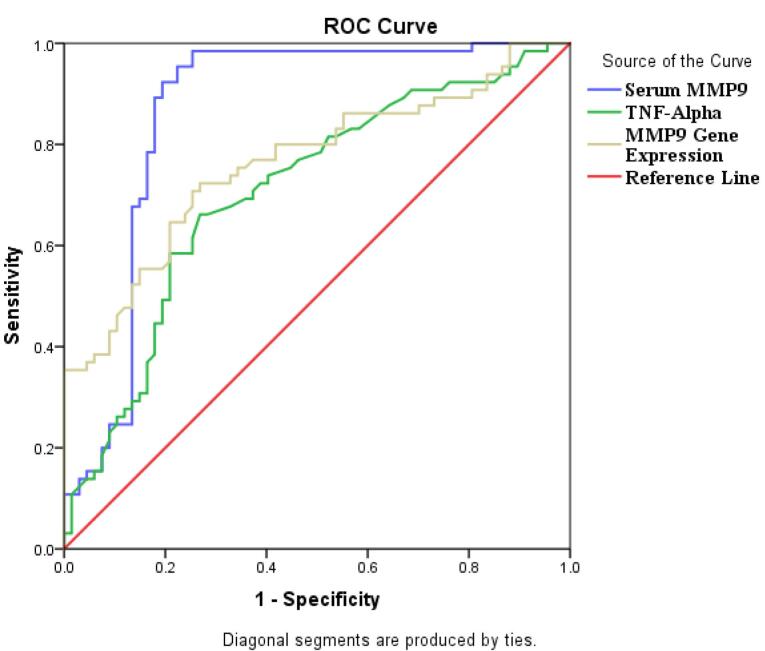
ROC curve analysis for MMP-9 gene, TNF-α and MMP-9 levels of CAD patients

 ROC curve analysis was carried out on MMP-9 gene expression, MMP-9 and TNF-α levels, which revealed these factors had a high discriminatory power for detecting CAD.ROC analysis revealed the following Area under curve (AUC) for the MMP-9 gene, MMP-9 and TNF-α levels (AUC = 0.763, 95% CI: 0.682, 0.845, AUC = 0.863, 95% CI: 0.792, 0.934, 0.707, 95% CI: 0.618, 0.796; all *P *value = 0.001, respectively) (Figure 5).

 According to Table 3, in controls, a significant positive association was observed between MMP-9 Levels with gene expression of MMP-9, and BMI with TIMP1 gene expression (all *P* < 0.05). moreover, a positive correlation between MMP-9 and IL-6 levels was detected (*P* = 0.01).

**Table 3:** Pearson analysis of correlation between variables in controls

	**TIMP-1 gene expression**	**MMP-9 Levels**	**MMP-9 gene expression**
TIMP-1 gene expression	1	r = 0.052	r = 0.162
MMP-9 Levels	r = 0.052	1	r = 0.274^*^
MMP-9 gene expression	r = 0.162	r = 0.274^*^	1
Age(years)	r = 0.081	r = -00.016	r = -0.164
Gender (F/M)	r = -0.100	r = 0.148	r = 0.241^*^
BMI Kg/hight^2^	r = 0.282^*^	r = -0.038	r = -0.161
SBP ( mm Hg)	r = 0.144	r = -0.062	r = -0.197
DBP (mm Hg)	r = 0.150	r = -0.065	r = -0.062
FBS (mg/dl)	r = -0.072	r = -0.187	r = -0.107
TG (mg/dl)	r = 0.085	r = 0.154	r = 0.052
TC (mg/dl)	r = -0.184	r = 0.017	r = ‒ 0.256^*^
LDL-C (mg/dl)	r = -0.137	r = -0.036	r = -0.202
HDL-C (mg/dl)	r = -0.106	r = -0.051	r = -0.052
Vitamin D (ng/ml)	r = 0.110	r = 0.042	r = 0.124
TNF-α ( pg /ml )	r = -0.076	r = 0.084	r = 0.031
IL-6 (pg /ml)	r = -0.066	r = 0.251^*^	r = 0.117
Zn^+2^ ( µg/dl )	r = 0.111	r = -0.086	r = 0.113
Ca^+2^ (mg/dl)	r = -0.027	r = 0.008	r = -0.106

*the Pearson correlation analysis value “Correlation coefficient r” was used*) *P* value < 0.01 MMP-9 = Metalloproteinase-9, TIMP-1 = Tissue Inhibitor Metalloproteinase-1, SBP = Systolic blood pressure, DBP = Diastolic blood pressure Lipid profile = TC: total cholesterol, TG: triglyceride, LDL-C: low density lipoprotein, HDL-C; high density lipoprotein, *P*<0.05 is statistically significant.

 In patients with CAD, significant positive correlation was found between TIMP-1 gene expression with MMP-9 levels and PBMC gene expression of MMP-9 (*P* = 0.049 and *P* = 0.001 respectively) (Table 4). Additionally, Additionally, MMP-9 significantly correlated with IL-6, TNF-α, LDL-C levels, and DBP (*P* = 0.007, *P* = 0.003, *P* = 0.023, *P* = 0.040 respectively), while a negative significant association was observed between gene expression of TIMP-1 and IL-6 levels (*P* = 0.008).

**Table 4:** Pearson analysis for correlation between variables in CAD patients

	**TIMP1 gene expression**	**MMP-9 Levels**	**MMP-9 gene expression**
TIMP-1 gene expression	1	r = 0.223^*^	r = 0.370^**^
MMP-9 Levels	r = 0.223^*^	1	r = 0.394^**^
MMP-9 gene expression	r = 0.370^**^	r = 0.394^**^	1
Age (years)	r = -0.231^*^	r = 0.032	r = -0.039
Gender (F/M)	r = -0.170	r = 0.052	r = -0.022
BMI (Kg/hight^2^)	r = -0.032	r = -0.030	r = 0.057
SBP (mmHg)	r = -0.024	r = 0.151	r = -0.026
DBP (mm Hg)	r = -0.159	r = 0.233^*^	r = -0.133
FBS (mg/dl)	r = -0.165	r = -0.153	r = -0.122
TG (mg/dl)	r = -0.095	r = 0.095	r = -0.007
TC (mg/dl)	r = 0.056	r = 0.184	r = -0.047
LDL-C(mg/dl)	r = 0.068	r = 0.258^*^	r = -0.012
HDL-C (mg/dl)	r = -0.067	r = -0.061	r = -0.070
Vitamin D (ng/ml)	r = 0.098	r = -0.058	r = 0.056
TNF-α ( pg/ml)	r = -0.176	r = 0.408^**^	r = -0.056
IL-6 ( pg /ml )	r = ‒ 0.334^**^	r = 0.300^**^	r = -0.182
Zn^2+^ (( µg/dl )	r = 0.123	r = -0.126	r = 0.108
Ca^2+^ (mg/dl)	r = -0.044	r = -0.088	r = 0.025

MMP9 = Metalloproteinase-9, TIMP-1 = Tissue inhibitor Metalloproteinase 1, SBP = Systolic blood pressure, DBP = Diastolic blood pressure *the Pearson correlation analysis value “Correlation coefficient r” was used in bold that indicated the significance of analysis*) *P* Value < 0.01, ** *P* Value < 0.001 *P*<0.05 is statistically significant.

 As it shown in Figure 3, TNF-α and IL-6 levels of CAD patients were more than controls significantly (all *P* < 0.01)

 As shown in Figure 4 and Table 2, TIMP-1 gene expression no differ between CAD patients and controls.

Figure 3 shows that the gene expression of MMP-9 and the MMP-9 levels in CAD patients were considerably higher than those in the control group (all *P* < 0.01).

## Discussion

 Accumulating evidence has revealed that inflammatory cytokines of IL-6 and TNF-α contribute significantly to developing atherosclerosis and CAD.^22-25^ In this study, serum levels of cytokines of IL-6 and TNF-α were greatly higher than controls (*P* < 0.05). In line with our results, Wen et al. reported in patients with acute aortic dissection (AD) levels of IL-6, TNF-α and CRP significantly increased than control, although serum MMP-9 significantly increased in Chronic aortic dissection than in AD patients and controls.^25^ Their results indicated a gradual increase in TNF-α levels leads to MMP-9 upregulation in neutrophils and macrophages. Thus, a noteworthy link between TNF-α levels and the AD progression confirmed that inflammatory response has a critical role in MMP-9 activation and other inflammatory responses.^24^ In ST-segment elevation myocardial infarction (STEMI) patients, it revealed that levels of sCD40L, TIMP-1, IL-6 and MMP-9 were significantly elevated relative to controls, and a significant correlation was detected between IL-6 and Troponin-I (TnI).^26-28^ Furthermore, activation of CD40L and CD40 has a central role in recruiting inflammatory cells, aggravating local micro-inflammation and synthesis of IL-6 and TNF-α.^26-28^

 Our study revealed that in CAD patients’ MMP-9 serum levels and gene expression were significantly higher than in the control group. Furthermore, MMP-9 levels are significantly positively associated with PBMC MMP-9 gene expression in CAD patients. Our results agree with previous studies that have also revealed that MMP-1 and MMP-9 levels increased in individuals with CAD.^9,22^ However, contrary to our finding, some studies showed no significant difference between MMP-9 in CAD patients and controls.^29^ Our findings indicate no significant variation in TIMP-1 gene expression between both groups. However, we observed a significant positive correlation between TIMP-1 gene expression and the gene and serum concentration of MMP-9 in the CAD group (*P* < 0.05). Therefore, this association between both genes revealed a molecular mechanism for modulation of MMP-9 gene expression and activity (*P* < 0.05).^9-14^

 Jordakieva et al. suggested that patients who did not survive in the ICU unit had significantly higher levels of MMP-9 and TIMP-1 compared to those who did survive. They suggested that levels of MMP-9 and TIMP-1 could be recognized as prognostic biomarkers and independent predictors of survival in systemic inflammation and acute organ failure.^24^ There is significant debate on the role of TIMP-1 in heart disorders, which has led to conflicting reports. Though our study failed to find any differences in TIMP-1 gene expression in patients with CAD, patients with atrial fibrillation, cardiomyopathy or ischemic cardiomyopathy showed a significant decrease in TIMP-1 and TIMP-3 levels.^23^ Hence, this inconsistency is attributed to various types of heart diseases, types of sampling, the stage of disease, methodology and the studied population’s ethnicity.

 Interaction between MMP-9/TIMP-1 and cytokines is the critical feature of CAD progression, severity and predicting the formation of stable plaque or rupture in susceptible patients.^30,31^ It was found that There was a negative association between IL-6 levels and PBMC TIMP-1 gene expression (*P* = 0.003). Conversely, there is a positive correlation between IL-6 levels and MMP-9 (*P* = 0.007). In patients with HF, collagen metabolism is altered by MMPs/TIMPs and cytokine gene expression in different tissue, resulting in the upregulation of MMP-2, MMP-9, TIMP-1, TIMP-2, TIMP-3 and TIMP-4 genes.^28,29^ Thus, matrix remodeling and collagen types I and III imbalance are resulted in an increased degree of fibrosis in HF.^30^ The possible(s) molecular mechanism is enhanced IL-6 and TNF-α could up-regulate MMPs expression through activating of cyclooxygenase-2, PGE2 and microsomal prostaglandin synthase 1, culminating to JAK-STAT and MAPK (erk1/2)-signaling pathways activation.^30-32^

 Emerging literature has revealed that MMP-9 modulates lipid metabolism and regulates transcriptional responses to dietary cholesterol and LDL-C synthesis. Our results revealed that HDL-C significantly reduced in CAD patients than in controls, while lipid profile didn’t differ between groups. Furthermore, a significant correlation was observed between MMP-9 and LDL-C levels in our CAD patients, whereas an inverse correlation was observed between TC and MMP-9 gene expression in controls. Hernandez-Anzaldo et al. suggested that MMP-9 alters cholesterol metabolism through phospholipase A2 secretion and transcriptional responses in the liver.^33^ In diabetic patients with coronary heart disease (CHD) levels of MMP-9, Apo-Protein-E (APO-E), high sensitive CRP (hs-CRP) and lipid profile were increased, and a significant correlation was observed between MMP-6 with APO-E, hs-CRP, TC, TG, LDL and HDL levels.^34^ Accumulating data have proposed that oxidized LDL (oxLDL) is led to matrix degradation, plaque rapture and vascular damage in atherosclerosis by overexpression of MMP-9 and TIMP-1.^34-35^

 In CAD patients, hypertension induces vascular remodeling through modifications in ECM structure and composition, leading to vascular endothelium damage and muscle contractility, consequently affects blood pressure. However, in experimental for hypertension and atherosclerosis, MMP inhibition could attenuate arterial remodeling and human arterial remodeling.^34^ In the present study, there was a significant correlation between DBP and MMP-9 levels in CAD patients, whereas MMP-9 levels had no significant correlation with SBP. However, research indicates that heightened levels of MMP-9 may have a key role in the development of arterial stiffness and high blood pressure.^35-38^

 Additionally, MMP-2, MMP-9, and TIMP-1 levels tend to be higher in obese subjects with BMI over 24, although there was a controversy among researchers.^37,38^ The present study revealed that in CAD patients, there was an inverse significant association between BMI with PBMC TIMP-1 gene expression and MMP-9 levels (all *P* < 0.05). In line with our results, other studies reported that in subjects with obesity and overweight, levels of MMP-9 and TIMP-1 were increased and positively related to BMI.^37-39^

 According to NHANES III study, Vit-D deficiency was associated with CVD.^38^ Furthermore, in recent decades, a growing literature has revealed Vit-D suppresses the inflammatory response and NF-κB pathway, thereby attenuating the progression of CAD.^39,40^ In the present study, Vti-D levels of CAD patients were significantly lower than controls (*P* < 0.05). Interestingly, low Vit-D levels are linked with increased MMP-2 and MMP-9 expression, reduced TIMPs, elevated VCAM, and higher levels of pro-inflammatory cytokines IL-6, IL8, and TNF-α.^16,39-42^ Recent findings have suggested that Vit-D supplementation inhibits inflammation process, VCAM activation and NF-κB pathways, although these findings are still inconclusive and more research is needed to fully understand their effects.^41,42^

 Our ROC analysis results in CAD patients showed that the MMP-9 gene, MMP-9 and TNF-α levels can be valid biomarkers for evaluation and follow up of CAD. Therefore, increased MMP-9 gene expression, serum MMP-9 and TNF-α concentration in CAD patients could have key roles in pathophysiological state of vascular injury, susceptibility to plaque formation and atherosclerosis. It is suggested that MMP-9 and TNF-α levels have regulatory effects on ongoing inflammation, lipid metabolism and atherosclerosis.

 Taken together, it is worthy to note that the positive correlation between of MMP-9 and IL-6 levels and negative association between MMP-9 gene expression and total cholesterol levels in CAD patients could be considered as the novelty of present study. According to our ROC curve analysis result, serum MMP-9 concertation is suggested as a hallmark in CAD prognosis and monitoring.

 A small sample size, instability of total-RNA, and the variation in demographic and socioeconomic characteristics of subjects were major study limitations that might influence our findings.

## Conclusion

 The results indicated that the relationship between MMPs, TIMP1, and some cytokines could have a key role in the pathogenesis of atherosclerosis. Based on the present study high levels of TNF-α and IL-6 as well as vitamin D deficiency in studied participants could disturb the MMP-9/TIMP-1 balance and lipid metabolism, leading to plaque formation/ rupture in predisposed CAD patients.

## Acknowledgments

 The authors would like to thank all contributors who have achieved this study.

## Competing Interests

 Authors did not have any conflict of interest in this manuscript.

## Ethical Approval

 The study was approved by the Ethics committee of the Iran University of Medical Sciences (IR.IUMS.FMD.REC.1398.059).

## Funding

 Authors did not any special fund for performing this study.
